# Supplementary material for: Assigning mitochondrial localization of dual localized proteins using a yeast Bi-Genomic Mitochondrial-Split-GFP
Source: eLife. 2020 Jul 13;9:e56649. doi: 10.7554/eLife.56649 (PMC7358010; doi:10.7554/eLife.56649)
Supplement: Figure 1—source data 1. — Growth assay on permissive SC Glu plates, respiratory plates (SC Gly), and restrictive media lacking arginine (SC Glu -Arg) of the different strains used in the study (related to Figure 1B). Mitochondrial translation products in the MR6 and RKY112 strains (N = 2) monitored by pulse-chase labeling with radiolabeled [35S]methionine and [35S]cysteine (related to Figure 1D). [file elife-56649-fig1-data1.docx]

**Figure 1 source data 1.**
