# Supplementary material for: Assigning mitochondrial localization of dual localized proteins using a yeast Bi-Genomic Mitochondrial-Split-GFP
Source: eLife. 2020 Jul 13;9:e56649. doi: 10.7554/eLife.56649 (PMC7358010; doi:10.7554/eLife.56649)
Supplement: Figure 1—source data 2. [file elife-56649-fig1-data2.docx]

**Figure 1–source data 2.**
