# Supplementary figures and images for: Assigning mitochondrial localization of dual localized proteins using a yeast Bi-Genomic Mitochondrial-Split-GFP
Source: eLife. 2020 Jul 13;9:e56649. doi: 10.7554/eLife.56649 (PMC7358010; doi:10.7554/eLife.56649)

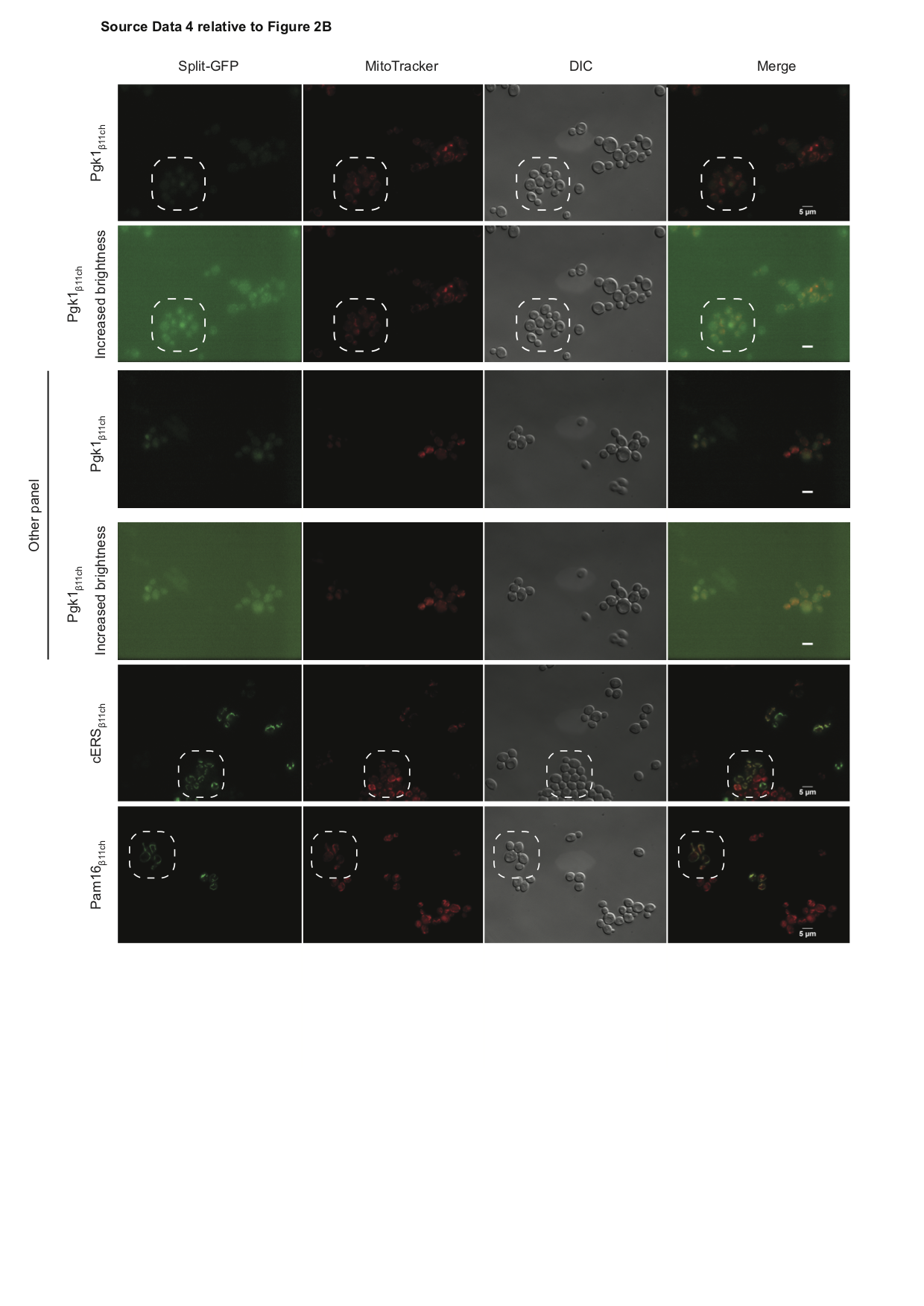


**Figure 2–source data 1.**

Supplement: Figure 2—source data 1. — The micrograph of the BiG Mito-Split-GFP expressing Pgk1β11ch which is magnified in Figure 2B is presented here with adjusted or enhanced contrast settings. A new panel of the BiG Mito-Split-GFP expressing Pgk1β11ch was added with enhanced or adjusted contrast settings. [file elife-56649-fig2-data1.docx]
