# Supplementary material for: Assigning mitochondrial localization of dual localized proteins using a yeast Bi-Genomic Mitochondrial-Split-GFP
Source: eLife. 2020 Jul 13;9:e56649. doi: 10.7554/eLife.56649 (PMC7358010; doi:10.7554/eLife.56649)
Supplement: Figure 5—source data 1. — Antibodies used for immunoblotting are indicated below WBs. Loading controls correspond to gels stained with the stain-free procedure. [file elife-56649-fig5-data1.docx]

**Figure 5–source data 1.**
