# Supplementary material for: Assigning mitochondrial localization of dual localized proteins using a yeast Bi-Genomic Mitochondrial-Split-GFP
Source: eLife. 2020 Jul 13;9:e56649. doi: 10.7554/eLife.56649 (PMC7358010; doi:10.7554/eLife.56649)
Supplement: Supplementary file 1. — The GFPβ1-10 sequence is in gray background and has been codon-optimized to be expressed by S. cerevisiae mitochondrial translation machinery. [file elife-56649-supp1.docx]

5’***GGATCC***ATAGTCCGGCCCGCCCCCCCCGCGGGGCGGACCCCAAAGGAGGAGTAATAAAAATTATTAAATACAAATATTATATATATATAATTCATTATATATATATATATATAATAATTAATCTTATTTTTTTATATATTTATTTATATATCTATTTATATTTTATATATATTTATTTATATATCTAAGGGGTTCGGTCCCTCCCCCCGTAAGTATAATATACGGGGGTGGGTCCCTCACTATTTATATTTTTATTTTATATATTTTATATATTTATAAATAAAGTATAATAAGATATAATTATGATTAATTATTTATAAGTTATAGTTTTATAAATTTATAATTATT**ATG**TCAAAAGGTGAAGAATTATTCACAGGTGTAGTTCCTATTTTAGTAGAATTAGATGGAGATGTTAATGGTCATAAATTTTCTGTAAGAGGTGAAGGTGAAGGAGATGCTACAATTGGTAAATTAACTTTAAAATTCATTTGTACAACTGGTAAATTACCTGTACCATGGCCTACATTAGTTACAACTTTAACTTATGGTGTACAATGTTTTTCAAGATATCCAGATCATATGAAACAACATGATTTCTTTAAATCAGCTATGCCTGAAGGTTATGTTCAAGAAAGAACAATTTCTTTTAAAGATGATGGTAAATATAAAACAAGAGCAGTAGTTAAATTTGAAGGAGATACTTTAGTAAATAGAATTGAATTAAAAGGTACAGATTTTAAAGAAGATGGTAATATTTTAGGTCATAAATTAGAATATAATTTTAATTCACATAATGTATATATTACTGCTGATAAACAAAAGAATGGTATTAAAGCAAATTTTACAGTAAGACATAATGTTGAAGATGGTTCTGTTCAATTAGCAGATCATTATCAACAAAATACTCCAATTGGAGATGGTCCAGTATTATTACCTGATAATCATTACTTATCAACACAAACTGTTTTATCTAAAGATCCTAATGAAAAATCAGGTCATCATCATCATCATCAT**TAA**ATTATAAAATAAAATTATAAAATAAAATAATTTACATATGGAGTATTAAACTATAATAAATACAATATACCCCATCCCCCCCTTTTAATAATATTCTTTTATCTAATAAAATATTTATTTATTAATATTATTATTATCTTCTTCAAGGACTTATTTAATATATTTAATAACTTATTATACTTATTTATATTTATAATTAATACAAATATATTATTAATCTTACTCCTTCGGAGTTCGGCCCCCCATAAGGGGGGGACCTCACTCCTTCCCCACTGCACTGGATGCGGGGA***GAATTC*** 3’

**Supplementary file 1.**
