# Supplementary material for: Assigning mitochondrial localization of dual localized proteins using a yeast Bi-Genomic Mitochondrial-Split-GFP
Source: eLife. 2020 Jul 13;9:e56649. doi: 10.7554/eLife.56649 (PMC7358010; doi:10.7554/eLife.56649)
Supplement: Supplementary file 2. — The use of each oligo is described in the Materials and methods section. [file elife-56649-supp2.docx]

| **Oligonucleotide name** | **Sequence** |
| --- | --- |
| oATP6-1 | 5’ TAATATACGGGGGTGGGTCCCTCAC 3’ |
| oATP6-2 | 5’ GTATGATTCCATACTCATTTGC 3’ |
| oATP6-4 | 5’ GCAAATGAGTATGGAATCATAC 3’ |
| oATP6-10 | 5’ GGGCCGAACTCCGAAGGAGTAAG 3’ |
| oXFP-lw | 5’ CCTGTGAATAATTCTTCACC 3’ |
| oXFP-pr | 5’ CAGGTCATCATCATCATC 3’ |
| o5`UTR2 | 5’ CCATCTCCATCTGTAAATCCTAC 3’ |
| o5`UTR1 | 5’ GAAGCGGGAATCCCGTAAGG 3’ |

**Supplementary file 2.**
