# Supplementary material for: Assigning mitochondrial localization of dual localized proteins using a yeast Bi-Genomic Mitochondrial-Split-GFP
Source: eLife. 2020 Jul 13;9:e56649. doi: 10.7554/eLife.56649 (PMC7358010; doi:10.7554/eLife.56649)
Supplement: Supplementary file 3. — The primers in black and blue were used for Gateway and Gibson cloning methods respectively (see Material and methods section). [file elife-56649-supp3.docx]

| **Standard name** | **Systematic Name** | **Protein** |  | **Primers used for PCR amplification** | | |
| --- | --- | --- | --- | --- | --- | --- |
|  |  |  |  | **Fw** |  | **Rv** |
|  | | | | | | |
| **Primers used to amplify genes for cloning to the pAG414pGPD_β11ch_ for genomic integration at the TRP1 locus** | | | | | | |
|  | | | | | | |
| *ALA1* | YOR335C | _cyte_cARS |  | ACAAGTTTGTACAAAAAAGCAGGCTTCATGACGATCGGTGATAAGC |  | ACTTTGTACAAGAAAGCTGGGTCTGGTCTTAATCTCTTTGGATCTC |
|  |  | _mte_cARS |  | gaacttagtttcgacggattctagaactagtACGACGTCAACTACCGG |  | TGCACGtaagcttgatatcgaattcctgcagCCAATGGAAAGCTTCTCCTTGAAC |
| *CRS1* | YNL247W | N_100_cCRS |  | agtttcgacggattctagaactagtATGAATATCTTCATAAAAGCCCTG |  | taagcttgatatcgaattcctgcagCCCACAAATTGCACGTCGTAAC |
| *DPS1* | YLL018C | cDRS |  | ACAAGTTTGTACAAAAAAGCAGGCTTCATGTCTCAAGACGAAAATATTGTC |  | ACTTTGTACAAGAAAGCTGGGTCTGGTCTTAATCTCTTTGGATCTC |
| *GUS1* | YGL245W | cERS |  | ACAAGTTTGTACAAAAAAGCAGGCTTCATGCCATCTACCTTGACTATTAATG |  | ACTTTGTACAAGAAAGCTGGGTCTTTCTTTGCACCATACTTGTTG |
|  |  | N_30_cERS |  | ACAAGTTTGTACAAAAAAGCAGGCTTCATGCCATCTACCTTGACTATTAATG |  | ACTTTGTACAAGAAAGCTGGGTCAGCTAACGCATTCACAATACG |
|  |  | N_70_cERS |  | ACAAGTTTGTACAAAAAAGCAGGCTTCATGCCATCTACCTTGACTATTAATG |  | ACTTTGTACAAGAAAGCTGGGTCACCATTATCGAAGATGGCG |
|  |  | N_200_cERS |  | ACAAGTTTGTACAAAAAAGCAGGCTTCATGCCATCTACCTTGACTATTAATG |  | ACTTTGTACAAGAAAGCTGGGTCACCCATTTTGGCATCTGG |
|  |  | ∆N_30_cERS |  | ACAAGTTTGTACAAAAAAGCAGGCTTCATGAACTCCATAGCTATTAAGTTGG |  | ACTTTGTACAAGAAAGCTGGGTCTTTCTTTGCACCATACTTGTTG |
|  |  | ∆N_200_cERS |  | ACAAGTTTGTACAAAAAAGCAGGCTTCATGGAAGTCGTCACTCGTTTCC |  | ACTTTGTACAAGAAAGCTGGGTCTTTCTTTGCACCATACTTGTTG |
| *FRS1* | YLR060W | cFRS1 |  | ACAAGTTTGTACAAAAAAGCAGGCTTCATGCCTACCGTCTCCG |  | ACTTTGTACAAGAAAGCTGGGTCTAGGAAGACTTCGGCATTAAC |
| *FRS2* | YFL022C | cFRS2 |  | gaacttagtttcgacggattctagaactagtATGTCTGACTTCCAATTAGAAATTC |  | TGCACGtaagcttgatatcgaattcctgcagCCTTCGTACAAGTCTTCGTCC |
| *GRS1* | YBR121C | _cyte_cGRS1 |  | ACAAGTTTGTACAAAAAAGCAGGCTTCATGAGTGTAGAAGATATCAAGAAGG |  | ACTTTGTACAAGAAAGCTGGGTCGTCAGTTTCAGCTTCAGC |
|  |  | _mte_cGRS1 |  | gaacttagtttcgacggattctagaactagtTTGTCGTTCTTCAATATCAGTAGAC |  | TGCACGtaagcttgatatcgaattcctgcagCCGTCAGTTTCAGCTTCAGCTTC |
| *GRS2* | YPR081C | cGRS2 |  | gaacttagtttcgacggattctagaactagtATGCCGTTAATGTCCAATTCG |  | TGCACGtaagcttgatatcgaattcctgcagCCTATCTTAACAGGCGACAGTCC |
| *HTS1* | YPR033C | _mte_cHRS |  | ACAAGTTTGTACAAAAAAGCAGGCTTCATGCTTAGTAGATCACTAAATAAAGTAG |  | ACTTTGTACAAGAAAGCTGGGTCTAATCCTTTAATTAAACGAGTGACC |
|  |  | _cyte_cHRS |  | gaacttagtttcgacggattctagaactagtATGTCATCCGCTACCGC |  | TGCACGtaagcttgatatcgaattcctgcagCCTAATCCTTTAATTAAACGAGTGACC |
| *ILS1* | YBL076C | cIRS |  | ACAAGTTTGTACAAAAAAGCAGGCTTCATGTCCGAGAGTAACGCAC |  | ACTTTGTACAAGAAAGCTGGGTCTAATTTGAACACTTTTAATTTGAAAATGG |
| *KRS1* | YDR037W | cKRS |  | ACAAGTTTGTACAAAAAAGCAGGCTTCATGTCTCAACAAGATAATGTCAAAG |  | ACTTTGTACAAGAAAGCTGGGTCATTTTCTTCTTCCTTTTTGACTTCC |
| *CDC60* | YPL160W | cLRS |  | ACAAGTTTGTACAAAAAAGCAGGCTTCATGTCTTCTGGTTTGGTCTTAG |  | ACTTTGTACAAGAAAGCTGGGTCAATATTTTGGAAGACAACACCTG |
| *MES1* | YGR264C | cMRS |  | ACAAGTTTGTACAAAAAAGCAGGCTTCATGTCTTTCCTCATTTCCTTTG |  | ACTTTGTACAAGAAAGCTGGGTCCACTTGTTGACCACCATATTTG |
| *DED81* | YHR019C | cNRS |  | ACAAGTTTGTACAAAAAAGCAGGCTTCATGTCATCTTTGTACATTAAGGAGG |  | ACTTTGTACAAGAAAGCTGGGTCTGGCTTACATCTACCGCTG |
|  |  |  |  |  |  |  |
| *GLN4* | YOR168W | cQRS |  | ACAAGTTTGTACAAAAAAGCAGGCTTCATGTCTTCTGTAGAAGAATTGACTC |  | ACTTTGTACAAGAAAGCTGGGTCCTTGGAAGTTGCGTCCTTC |
| *RRS1* | YDR341C | cRRS |  | ACAAGTTTGTACAAAAAAGCAGGCTTCATGGCTAGCACAGCAAATATG |  | ACTTTGTACAAGAAAGCTGGGTCCATTCTTTCTACGGGAGTTAAAC |
| *SES1* | HDR023W | cSRS |  | ACAAGTTTGTACAAAAAAGCAGGCTTCATGTTGGACATCAACCAATTTATC |  | ACTTTGTACAAGAAAGCTGGGTCATTCTTCTTTTTCTTGTCTTTACTAGAG |
| *THS1* | YIL078W | cTRS |  | ACAAGTTTGTACAAAAAAGCAGGCTTCATGAGTGCTAGTGAAGCAG |  | ACTTTGTACAAGAAAGCTGGGTCAGCTAAGACGTTGTCACCTC |
| *VAS1* | YGR094W | _mte_cVRS |  | ACAAGTTTGTACAAAAAAGCAGGCTTCATGAATAAGTGGTTAAACACATTATC |  | ACTTTGTACAAGAAAGCTGGGTCCAATTTCAAACGCTTCAAGTTTTC |
|  |  | _cyte_cVRS |  | gaacttagtttcgacggattctagaactagtATGAGCGATCTTGATAATTTGCC |  | TGCACGtaagcttgatatcgaattcctgcagCCCAATTTCAAACGCTTCAAGTTTTC |
| *WRS1* | YOL097C | cWRS |  | ACAAGTTTGTACAAAAAAGCAGGCTTCATGAGCAACGACGAAACTG |  | ACTTTGTACAAGAAAGCTGGGTCCTTCTTTTCTTGCTTAGTTTTTGG |
| *TYS1* | YGR185C | cYRS |  | ACAAGTTTGTACAAAAAAGCAGGCTTCATGTCCTCTGCTGCCAC |  | ACTTTGTACAAGAAAGCTGGGTCCAATTTGGTTTCCTCTAGTTTCG |
| *PAM16* | YJL104W | Pam16 |  | ACAAGTTTGTACAAAAAAGCAGGCTTCATGGCTCACAGGGC |  | ACTTTGTACAAGAAAGCTGGGTCCTGATTGCTGCTTGCAC |
| *PGK1* | YCR012W | Pgk1 |  | ACAAGTTTGTACAAAAAAGCAGGCTTCATGTCTTTATCTTCAAAGTTGTCTG |  | ACTTTGTACAAGAAAGCTGGGTCTTTCTTTTCGGATAAGAAAGCAAC |
| *ATP4* | YPL078C | Atp4 |  | ACAAGTTTGTACAAAAAAGCAGGCTTCATGAGCATGAGTATGGGTG |  | ACTTTGTACAAGAAAGCTGGGTCCTTCAATTTAGAAAGCAATTGTTCA |
| *Ago2* |  | *Mmu*Ago2 |  | gaacttagtttcgacggattctagaactagtatggacatccccaaaattgac |  | GCACGtaagcttgatatcgaattcctgcagCCagcaaagtacatggtgcg |
| *AGO2* |  | *Hsa*Ago2 |  | gaacttagtttcgacggattctagaactagtatggacatccccaaaattgac |  | GCACGtaagcttgatatcgaattcctgcagCCagcaaagtacatggtgcg |
| *At5g26710* |  | *Ath*cERS |  | ACAAGTTTGTACAAAAAAGCAGGCTTCATGGATGGGATGAAGCTTTC |  | ACTTTGTACAAGAAAGCTGGGTCCCTTAGCGGCTCTTCC |
| *OVA3* |  | *Ath*mt/chlERS |  | ACAAGTTTGTACAAAAAAGCAGGCTTCATGGCGAGCCTTGTCTAC |  | ACTTTGTACAAGAAAGCTGGGTCCGGTTGATACTGTGGCTG |
| **Primers used to amplify genes for cloning to the pAG304pGPD_β11ch_ for genomic integration at the *TRP1* locus** | | | | | | |
|  | | | | | | |
| *GUS1* | YGL245W | cERS |  | tatagggcgaattggagctcTCCGATATGTTCGCAACTTG |  | actaattacatgactcgagTCAGGTACCAGTAATACCAGC |
| *PGK1* | YCR012W | Pgk1 |  | tatagggcgaattggagctctcgagtttatcattatcaatactcgc |  | gtgacataactaattacatgactcgagTCAGGTACCAGTAATACCAGC |
| *PAM16* | YJL104W | Pam16 |  | tatagggcgaattggagctctcgagtttatcattatcaatactcgc |  | gtgacataactaattacatgactcgagTCAGGTACCAGTAATACCAGC |
| **Primers used to amplify the WT coding sequence of *ADE2* for restauration of adenine auxotrophy** | | | | | | |
|  | | | | | | |
| *ADE2* | YOR128C | Ade2 |  | TGACAAATGACTCTTGTTGCATGGC |  | AATTATTCCTTGCTTCTTGTTACTGG |

**Supplementary file 3.**
