# Supplementary material for: Assigning mitochondrial localization of dual localized proteins using a yeast Bi-Genomic Mitochondrial-Split-GFP
Source: eLife. 2020 Jul 13;9:e56649. doi: 10.7554/eLife.56649 (PMC7358010; doi:10.7554/eLife.56649)
Supplement: Supplementary file 4. [file elife-56649-supp4.docx]

| **Protein** | **Gene** | **Plasmid** | **expected MW (kDa)** | **transformed in** |
| --- | --- | --- | --- | --- |
| _cyte_cARS _β11ch_ | *ALA1* | pAG414pGPD *ALA1* _β11ch_ | 118 | BiG Mito-Split-GFP  *MATa his3-11,15 trp1-1 leu2-3,112 ura3-1 CAN1 arg8::HIS3* |
| _mte_cARS _β11ch_ | *ALA1-25_ACG_* | pAG414pGPD *ALA1-25_ACG_* _β11ch_ | 119 |  |
| cDRS _β11ch_ | *DPS1* | pAG414pGPD *DPS1* _β11ch_ | 74 |  |
| cERS _β11ch_ | *GUS1* | pAG414pGPD *GUS1* _β11ch_ | 91 |  |
|  | *GUS1* | pAG414pGUS1 *GUS1* _β11ch_ | 91 |  |
| N_30_cERS _β11ch_ | _N30_*GUS1* | pAG414pGPD *_N30_GUS1* _β11ch_ | 13 |  |
| ∆N_30_cERS _β11ch_ | _∆N30_*GUS* | pAG414pGPD *_∆N30_GUS1* _β11ch_ | 87 |  |
| N_70_cERS _β11ch_ | _N70_*GUS1* | pAG414pGPD *_N70_GUS1* _β11ch_ | 18 |  |
| N_200_cERS _β11ch_ | _N200_*GUS1* | pAG414pGPD *_N200_GUS1* _β11ch_ | 32 |  |
| ∆N_200_cERS _β11ch_ | _∆N200_*GUS1* | pAG414pGPD *_∆N200_GUS1* _β11ch_ | 69 |  |
| cFRS1 _β11ch_ | *FRS1* | pAG414pGPD *FRS1* _β11ch_ | 76 |  |
| cFRS2 _β11ch_ | *FRS2* | pAG414pGPD *FRS2* _β11ch_ | 66 |  |
| _cyte_cGRS1 _β11ch_ | *GRS1* | pAG414pGPD *GRS1* _β11ch_ | 86 |  |
| _mte_cGRS1 _β11ch_ | *GRS1-23_TTG_* | pAG414pGPD *GRS1-23_TTG_* _β11ch_ | 87 |  |
| cGRS2 _β11ch_ | *GRS2* | pAG414pGPD *GRS2* _β11ch_ | 80 |  |
| _cyte_cHRS _β11ch_ | *HTS1∆20_ATG_* | pAG414pGPD *HTS1∆20_ATG_* _β11ch_ | 67 |  |
| _mte_cHRS _β11ch_ | *HTS1* | pAG414pGPD *HTS1* _β11ch_ | 70 |  |
| cIRS _β11ch_ | *ILS1* | pAG414pGPD *ILS1* _β11ch_ | 133 |  |
| cKRS _β11ch_ | *KRS1* | pAG414pGPD *KRS1* _β11ch_ | 78 |  |
| cLRS _β11ch_ | *CDC60* | pAG414pGPD *CDC60* _β11ch_ | 134 |  |
| cMRS _β11ch_ | *MES1* | pAG414pGPD *MES1* _β11ch_ | 96 |  |
| cNRS _β11ch_ | *DED81* | pAG414pGPD *DED81* _β11ch_ | 72 |  |
| cQRS _β11ch_ | *GLN4* | pAG414pGPD *GLN4* _β11ch_ | 103 |  |
| cRRS _β11ch_ | *RRS1* | pAG414pGPD *RRS1* _β11ch_ | 80 |  |
| cSRS _β11ch_ | *SES1* | pAG414pGPD *SES1* _β11ch_ | 64 |  |
| cTRS _β11ch_ | *THS1* | pAG414pGPD *THS1* _β11ch_ | 95 |  |
| _mte_cVRS _β11ch_ | *VAS1* | pAG414pGPD *VAS1* _β11ch_ | 136 |  |
| _cyte_cVRS _β11ch_ | *VAS1∆46_ATG_* | pAG414pGPD *VAS1∆46_ATG_* _β11ch_ | 129 |  |
| cWRS _β11ch_ | *WRS1* | pAG414pGPD *WRS1* _β11ch_ | 60 |  |
| cYRS _β11ch_ | *TYS1* | pAG414pGPD *TYS1* _β11ch_ | 54 |  |
| Pam16 _β11ch_ | *PAM16* | pAG414pGPD *PAM16* _β11ch_ | 27 |  |
|  | *PAM16* | pAG304pGPD *PAM16* _β11ch_ | 27 |  |
| Atp4 _β11ch_ | *ATP4* | pAG414pGPD *ATP4* _β11ch_ | 37 |  |
| Pgk1 _β11ch_ | *PGK1* | pAG414pGPD *PGK1* _β11ch_ | 55 |  |
|  | *PGK1* | pAG304pGPD *PGK1* _β11ch_ | 55 |  |
| N_100_cCRS _β11ch_ | *_N100_CRS1* | pAG414pGPD *_N100_CRS1* _β11ch_ | 21 |  |
| *Ath*cERS _β11ch_ | *At5g26710* | pAG414pGPD *At5g26710* _β11ch_ | 93 |  |
| *Ath*mt/chlERS_β11ch_ | *OVA3* | pAG414pGPD *OVA3* _β11ch_ | 73 |  |
| *Mmu*Ago2 _β11ch_ | *AGO2* | pAG414pGPD *AGO2* _β11ch_ | 102 |  |
| *Hsa*Ago2 _β11ch_ | *AGO2* | pAG414pGPD *AGO2* _β11ch_ | 102 |  |

| **Proteins** | **Genes** | **Plasmids** | **expected MW (kDa)** | **transformed in** |
| --- | --- | --- | --- | --- |
| mtGatF_β1-10_ | *GTF1* | pRS415pADH *GTF1* _β1-10_ | 45 | BY4742  *MATα his3Δ1 leu2Δ0 lys2Δ0 ura3Δ0* |

**Supplementary file 4.**
