# Supplementary material for: Assigning mitochondrial localization of dual localized proteins using a yeast Bi-Genomic Mitochondrial-Split-GFP
Source: eLife. 2020 Jul 13;9:e56649. doi: 10.7554/eLife.56649 (PMC7358010; doi:10.7554/eLife.56649)
Supplement: Transparent reporting form [file elife-56649-transrepform.docx]

***eLife’s* transparent reporting form**

We encourage authors to provide detailed information *within their submission* to facilitate the interpretation and replication of experiments. Authors can upload supporting documentation to indicate the use of appropriate reporting guidelines for health-related research (see [EQUATOR Network](about:blank)), life science research (see the BioSharing Information Resource), or the [ARRIVE guidelines](about:blank) for reporting work involving animal research. Where applicable, authors should refer to any relevant reporting standards documents in this form.

If you have any questions, please consult our Journal Policies and/or contact us: [editorial@elifesciences.org](about:blank).

**Sample-size estimation**

- You should state whether an appropriate sample size was computed when the study was being designed
- You should state the statistical method of sample size computation and any required assumptions
- If no explicit power analysis was used, you should describe how you decided what sample (replicate) size (number) to use

Please outline where this information can be found within the submission (e.g., sections or figure legends), or explain why this information doesn’t apply to your submission:

As for classical yeast experiments, the sample size is always important. Cells were grown to early log-phase (0D600nm=0.8) which represents 12e6 cells for each biological replicates. This is why every experiment was done either with two or three replicates, sufficient to reflect a large population of cells.

This applies to Figures 1B, 1C, 1D, Figures 2B, 2C, 2E, Figures 3A, 3C, 3D, Figure 4B, Figures 5B, 5C, 5F, Figure 2-figure supplement 1 and Figure 3-figure supplement 1. The information about sample size can always be found in their corresponding figure legends.

The purpose of the BiG-Mito-Split GFP strain is not to compare the relative fluorescence intensities between GFP-positive strains, therefore we did not have to compute GFP fluorescence intensities and thus there was no need for statistics.

In Figure 2-source data 1, we show how cells presented in the above-mentioned figures were selected from each microscopy field replicate.

**Replicates**

- You should report how often each experiment was performed
- You should include a definition of biological versus technical replication
- The data obtained should be provided and sufficient information should be provided to indicate the number of independent biological and/or technical replicates
- If you encountered any outliers, you should describe how these were handled
- Criteria for exclusion/inclusion of data should be clearly stated
- High-throughput sequence data should be uploaded before submission, with a private link for reviewers provided (these are available from both GEO and ArrayExpress)

Please outline where this information can be found within the submission (e.g., sections or figure legends), or explain why this information doesn’t apply to your submission:

Experiments 1B, 1C, 1D has been repeated two times as biological replicates (differents clones were tested each time)

Experiments 2B, 2E has been repeated three times as biological replicates (differents clones were tested each time)

Experiment 2D is related to observations done in 2B and was done once

Experiments 3A and of Figure 3-supplement 1A were done three times in biological replicates each time and experiments 3C, 3D, 4B, 5B and 5E two times in biological replicates each time

Experiments 4A, 4C, 5B and 5E were repeated two times with biological replicates

Experiments in figure 1-figure supplement 2D and 2E was done on 4 biological replicates, and S2C on three biological replicates

All of these informations are available in their respective figure legends

**Statistical reporting**

- Statistical analysis methods should be described and justified
- Raw data should be presented in figures whenever informative to do so (typically when N per group is less than 10)
- For each experiment, you should identify the statistical tests used, exact values of N, definitions of center, methods of multiple test correction, and dispersion and precision measures (e.g., mean, median, SD, SEM, confidence intervals; and, for the major substantive results, a measure of effect size (e.g., Pearson's r, Cohen's d)
- Report exact p-values wherever possible alongside the summary statistics and 95% confidence intervals. These should be reported for all key questions and not only when the p-value is less than 0.05.

Please outline where this information can be found within the submission (e.g., sections or figure legends), or explain why this information doesn’t apply to your submission:

For the experiments done in 1C, an unpaired T-test was used with equal SD (two-tailed), using two independent biological replicates (N=2). P-value was 0.7456 (not significant). 95% confidence interval was -273.4 to 229.9, R squared=0.064.

This information is available in legend to figure 1C and Figure 1-source data 2

(For large datasets, or papers with a very large number of statistical tests, you may upload a single table file with tests, Ns, etc., with reference to sections in the manuscript.)

**Group allocation**

- Indicate how samples were allocated into experimental groups (in the case of clinical studies, please specify allocation to treatment method); if randomization was used, please also state if restricted randomization was applied
- Indicate if masking was used during group allocation, data collection and/or data analysis

Please outline where this information can be found within the submission (e.g., sections or figure legends), or explain why this information doesn’t apply to your submission:

Not relevant for this study

**Additional data files (“source data”)**

- We encourage you to upload relevant additional data files, such as numerical data that are represented as a graph in a figure, or as a summary table
- Where provided, these should be in the most useful format, and they can be uploaded as “Source data” files linked to a main figure or table
- Include model definition files including the full list of parameters used
- Include code used for data analysis (e.g., R, MatLab)
- Avoid stating that data files are “available upon request”

Please indicate the figures or tables for which source data files have been provided:

Source data have been provided for:

- Figure 1B, 1C, 1D
- Figure 2B, 2C, 2E
- Figure 3-figure supplement 1B
- Figure 4C
- Figure 5C, 5F
